# Treatment of immunoglobulin-resistant kawasaki disease: a Bayesian network meta-analysis of different regimens

**DOI:** 10.3389/fped.2023.1149519

**Published:** 2023-07-13

**Authors:** Yan Pan, Qihong Fan, Luoyi Hu

**Affiliations:** Department of Pediatrics, The First Affiliated Hospital of Yangtze University, Jingzhou, China

**Keywords:** immunoglobulin, resistant kawasaki disease, infliximab, IVIG, methylprednisolone

## Abstract

**Background:**

This study aimed to gather evidence from clinical trials on the efficacy and safety of the available treatments for intravenous immunoglobulin (IVIG)-resistant Kawasaki disease (KD) in children.

**Methods:**

This work adopted the Newcastle–Ottawa scale to analyse the quality of the enrolled articles. A network meta-analysis was performed using clinical trials that compared drugs used to treat IVIG-resistant KD. Aggregate Data Drug Information System software v.1.16.5 was employed to analyse whether infliximab, second IVIG infusions, and intravenous pulse methylprednisolone (IVMP) were safe and effective.

**Results:**

Ten studies, involving 704 patients with IVIG-resistant KD, were identified and analysed. Overall, infliximab exhibited remarkable antipyretic activity compared with the second IVIG infusions (2.46, 1.00–6.94). According to the drug rank, infliximab was the best option against IVIG-resistant KD. Regarding adverse effects, the infliximab group was more prone to hepatomegaly. A second IVIG infusion was more likely to result in haemolytic anaemia. IVMP treatment was more susceptible to bradycardia, hyperglycaemia, hypertension, and hypothermia. In addition, infliximab, IVMP, and the second IVIG infusions showed no significant differences in the risk of developing a coronary artery aneurysm (CAA).

**Conclusion:**

Infliximab was the best option against IVIG-resistant KD, and IVMP, infliximab, and second IVIG infusions have not significant differences of prevent CAA in patients with IVIG-resistant KD.

**Systematic Review Registration:**

Identifier: https://osf.io/3894y.

## Introduction

Kawasaki disease (KD) is an acute, self-limiting, systemic vascular inflammation mainly occurring in small arteries, particularly the coronary arteries (1, 2). In the acute stage, immunoglobulins administered at high doses may decrease coronary artery injury, but 15%–20% of such cases will develop intravenous immunoglobulin (IVIG)-resistant KD (3). According to the literature, coronary artery aneurysm (CAA) incidence is 9-fold higher in IVIG-resistant KD cases than that in IVIG-sensitive cases (4). IVIG-resistant KD may have an increased risk of coronary artery injury compared to IVIG-sensitive KD. Therefore, the risk of coronary artery injury and hospitalisation duration and costs are reduced if IVIG-resistant KD cases are detected, and appropriate treatment is administered prior to further IVIG therapy.

For febrile IVIG-resistant cases, no clear guidelines are available for treatment, which presents a typical challenge. Patients with IVIG-resistant KD should be treated with the second IVIG infusions (2 g/kg for 1 day). An alternative approach is either 30 mg/kg intravenous pulse methylprednisolone (IVMP; 30 mg/kg for 2–3 h once daily for 3 days) or infliximab (5 mg/kg for 1 day) (5). Infliximab is the drug of choice for treating IVIG-resistant KD (6). However, no uniform treatment guidelines are available, and many different treatments exist among diverse medical centres (7). In addition, drug-related adverse effects (AEs) remain unclear.

Several studies have investigated different drugs for treating IVIG-resistant KD. Previous meta-analyses showed that IVMP and infliximab exhibited higher efficacy than the second IVIG infusions (8, 9). However, the previous pairwise meta-analysis could only analyse two drugs. Network meta-analysis (NMA) can analyse multiple drugs based on clinical research. It has a high reference value for evaluating the advantages of interventions and can provide the best evidence for clinical decision-making. This study aimed to perform a systematic review and Bayesian NMA on paediatric patients reported in studies published in several databases over the past 15 years to investigate the efficacy and safety of different drug regimens for treating IVIG-resistant KD.

## Methods

The present study performed NMA and followed the Preferred Reporting Items for Systematic Reviews and Meta-Analysis guidelines extended to NMA (10). Additionally, this study utilised a population-intervention-comparison-outcome framework to include studies describing the treatment of IVIG-resistant KD. Our study protocols were registered in the OSF Registries (https://osf.io/3894y).

### Database search

Relevant databases, such as PubMed, Embase, ScienceDirect, ProQuest, ClinicalTrials.gov, ClinicalKey, Cochrane CENTRAL, and Web of Science, were comprehensively searched until 1 May, 2022 to identify relevant studies. The search strategy was approved by the review teams (LH and QF). Regarding the search strategy, the Medical Subject Headings used were (Mucocutaneous Lymph Node Syndrome OR Kawasaki disease) AND (methylprednisolone OR intravenous immunoglobulin/IVIG OR infliximab OR corticosteroids OR steroids OR glucocorticoids OR TNF blockers). No study design or language restrictions were imposed. Additionally, the reference lists of the enrolled studies were manually searched. Finally, two reviewers (LH and QF) reviewed the studies and extracted relevant data.

### Study quality assessment

This study used the Newcastle–Ottawa scale (NOS) to assess the quality of the enrolled observational studies. Typically, we judged the NOS statements on three aspects (selection, outcome, and comparability) involving eight items. The Cochrane Collaboration-recommended risk-of-bias approach was used to assess the quality of the randomised clinical trials. A final score of six or more stars was considered high quality.

### Selection criteria

Studies conforming to the criteria below were included: (a) patients with the diagnosis of KD in line with the Japanese diagnostic criteria, as well as common standards from the 2017 American Heart Association (i.e., IVIG resistance was defined as persistent or recrudescent fever [T ≥38.0 °C] at least 36 h after completion of the first IVIG infusion), (b) odds ratios (ORs) together with relevant 95% confidence intervals (CIs) regarding categorical variables or numbers and standard deviations could be obtained from the studies, and (c) statistical approaches were clearly described, and statistical analysis was conducted accordingly. The following studies were excluded: (a) studies with defects or low-quality (NOS score<six stars), (b) no ORs or 95% CIs could be obtained for categorical variables, and (c) reviews, duplicates, or unpublished literature.

### Statistical analyses

This study utilised NMA to analyse all enrolled articles. Moreover, Aggregate Data Drug Information System software v. 1.16.6 was used to compare the safety and effectiveness of diverse therapeutic agents (11). The Bayesian method was applied in the NMA, which made it possible to compare diverse treatments among different studies (12). We adopted a random-effects model with the Bayesian method through a Markov chain Monte Carlo simulation to obtain the combined effect sizes. We also drew a consistency model to analyse the outcomes assessed and determined the relative effect sizes of treatments based on ORs. Instead of the fixed-effects model, we utilised the random-effects model because it is suitable and conservative for interpreting interstudy variance. Residual deviance was also used to evaluate the goodness of fit of the models. To increase the accuracy of comparison effect sizes and appropriately explain the relationships of multiarm studies, this study constructed rank probabilities that involved every intervention in every outcome to draw conclusions for diverse outcomes of interest (13). Data were expressed with 95% CIs. Subsequently, diverse treatments were ordered based on the highest to the lowest probabilities.

## Result

### Study selection and description

**Figure 1:**
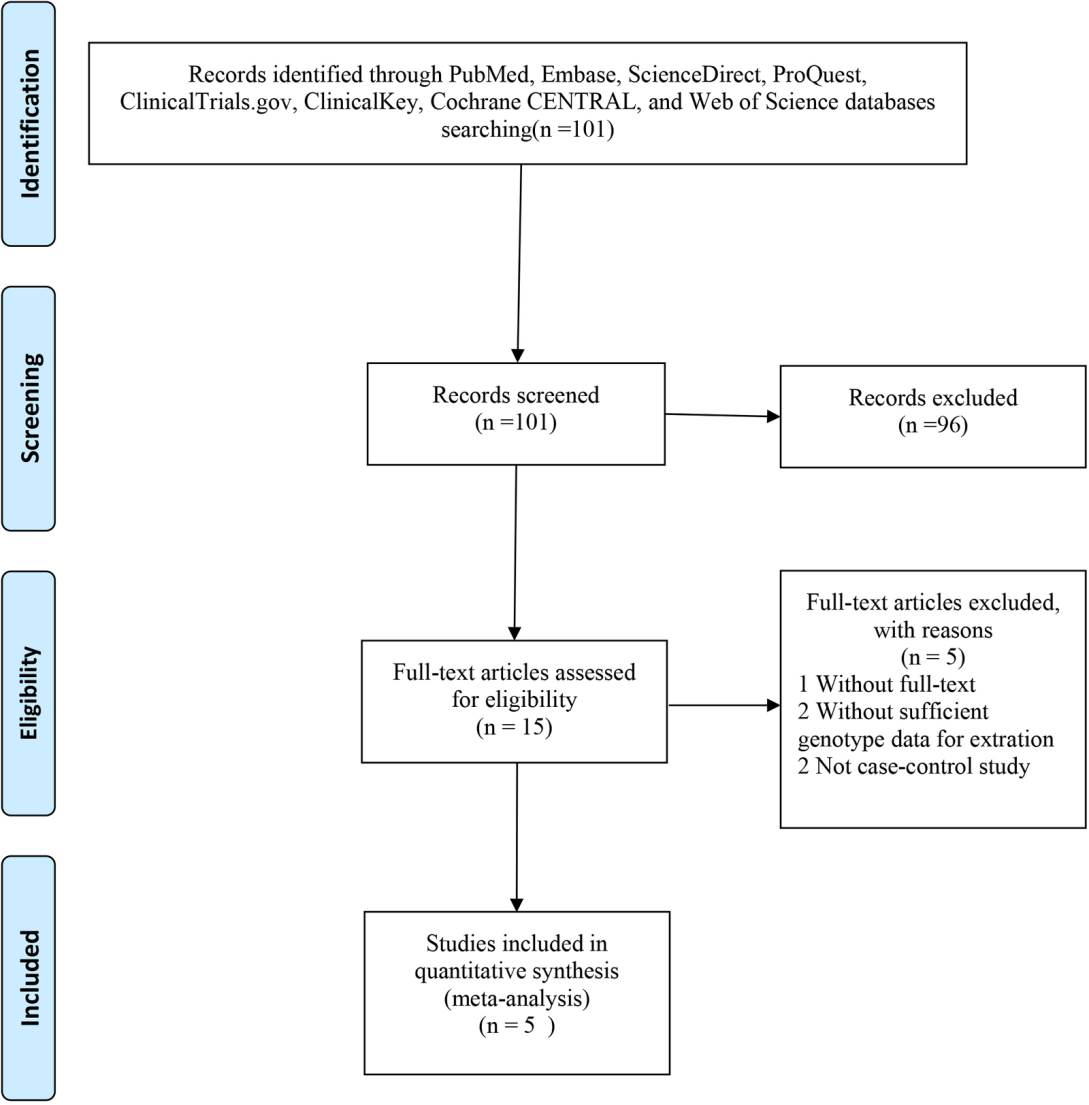
The flowchart of the included studies in the meta-analysis.

A total of 101 eligible articles were included (Figure 1). Of these, 96 did not meet the inclusion criteria and were not subjected to further examination. We excluded two publications because they did not provide detailed genotypic information. We also excluded two publications because they were not case-control studies. Furthermore, one publication was removed because the full text was not available. In line with our inclusion and exclusion criteria, this work selected 10 articles published between 2003 and 2021 (13–22). Among these, seven were randomised controlled trials (RCTs) (1, 15, 16, 20–22), and three were non-RCTs (14, 18, 19), as determined based on the Cochrane Handbook. The generation of random sequences was not utilised by Furakawa et al. and Teraguchi et al. since some patients were unwilling to receive IVMP and therefore received a second dose of IVIG (14, 19). Data from Son et al. were collected through a retrospective chart review, and all studies were rated as ≥six stars (high quality) (18). Baseline features on admission were comparable among the diverse treatments, such as age at fever onset, sex, race, duration between fever onset and diagnosis, and duration from the first treatment to retreatment. Five studies were conducted in Japan, three in America, and one each in China and Korea (Table 1).

**Table 1:** Study selection and subject characteristics of included studies in meta-analysis.

Author	Year	Country	Design	Patients		Initial treatment	Retreatment		Antipyretic effects		CAA		NOS
				Cases	Controls		Cases	Controls	Cases	Controls	Cases	Controls	
Son	2011	US	Non-RCT	20	86	IVIG	Infliximab	IVIG	17	71	/	/	6
Tremoulet	2014	US	RCT	98	97	IVIG	Infliximab	IVIG	87	86	25	20	9
Youn	2016	Korea	RCT	11	32	IVIG	Infliximab	IVIG	10	21	1	4	6
Masaaki	2018	Japan	RCT	16	15	IVIG	Infliximab	IVIG	12	5	1	3	8
Burns	2021	US	RCT	54	49	IVIG	Infliximab	IVIG	42	25	4	1	9
Miura	2008	Japan	RCT	7	8	IVIG	IVMP	IVIG	4	4	2	2	8
Furukawa	2008	Japan	Non-RCT	44	19	IVIG	IVMP	IVIG	34	12	5	2	6
Ogata	2009	Japan	RCT	13	14	IVIG	IVMP	IVIG	13	14	0	3	8
Teraguchi	2013	Japan	Non-RCT	14	27	IVIG	IVMP	IVIG	7	21	5	7	6
Wang	2020	China	RCT	40	40	IVIG	IVMP	IVIG	40	40	18	15	9

RCT, randomized controlled trial; NOS, newcastle-ottawa scale; CAA, coronary artery aneurysm.

### Antipyretic effects

**Figure 2:**
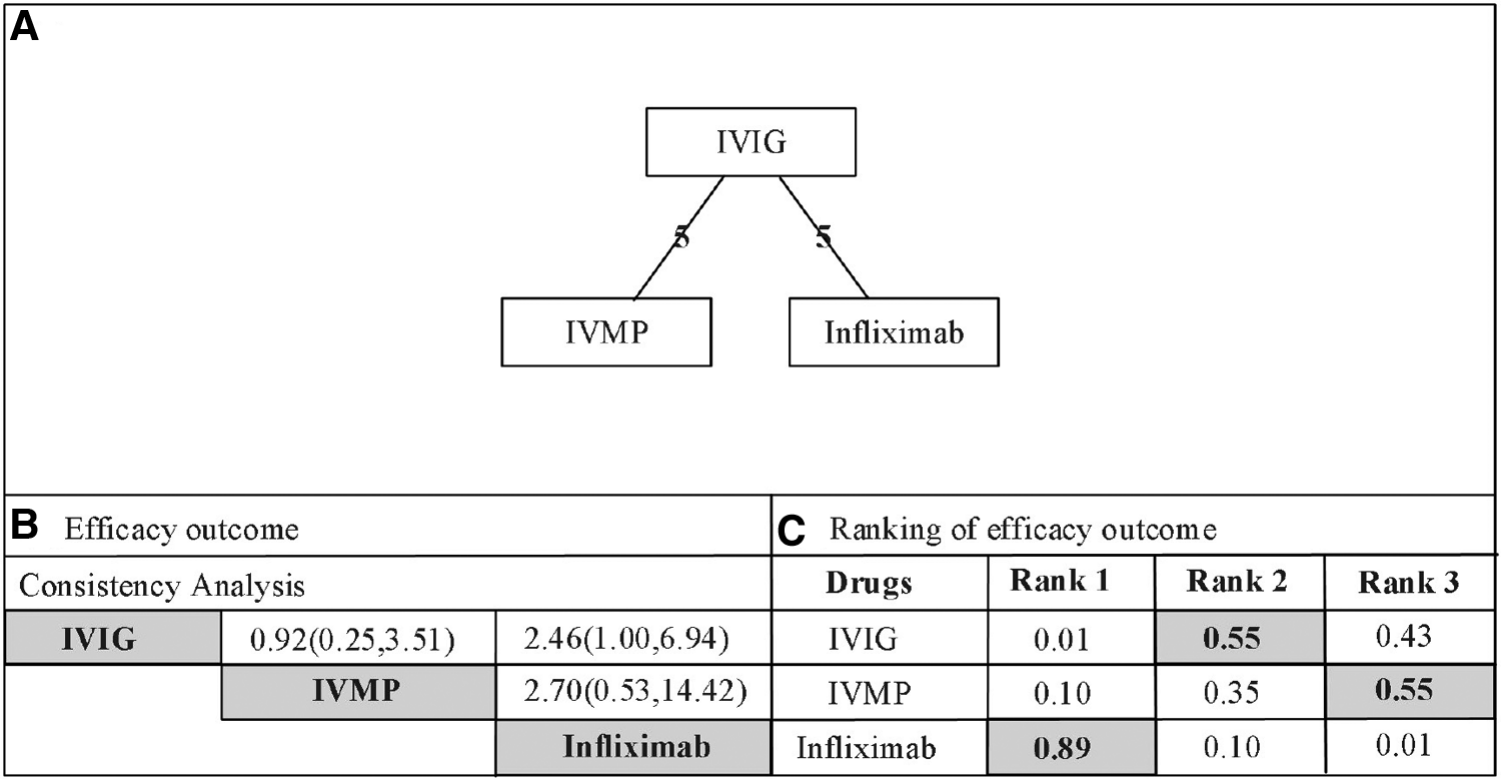
(**A**) Network of comparisons for efficacy outcome. The nodes (drugs) are represented by circles. The grey circles represent stimulant drug and the white circles represent non-stimulant drugs. The lines connecting each drug represent direct comparisons, while indirect ones were statistically estimated. The thickness of the line represents the amount of existing comparisons and the size of the circles (nodes) indicates the sample-size number. (**B**) Consistency analysis for the outcome of efcacy. Drugs are reported in alphabetical order. The values presented correspond to the mean diference (MD) associated with its credibility interval (CrI). When the CrI does not cross the 0 null line, there is a statistically signifcant diference between the treatments. Comparisons are made between a first drug (e.g. IVIG) and a second drug (e.g. IVMP) with presentation of the estimated value (0.92 [0.25-3.51]). An MD value of less than 0 demonstrates that the frst drug in the comparison is the more effective. An MD value greater than 0 indicates that the second drug in the comparison is more efective. The highlighted pictures presented statistical diferences. IVIG, intravenous immunoglobulin; IVMP, intravenous pulse methylprednisolone. (**C**) Rank probabilities of drugs. The values are given as the probability of each treatment occupying a position. Ranking 1 is the best therapy and the last one is the worst treatment for this outcome. IVIG, intravenous immunoglobulin; IVMP, intravenous pulse methylprednisolone.

**Figure 3:**
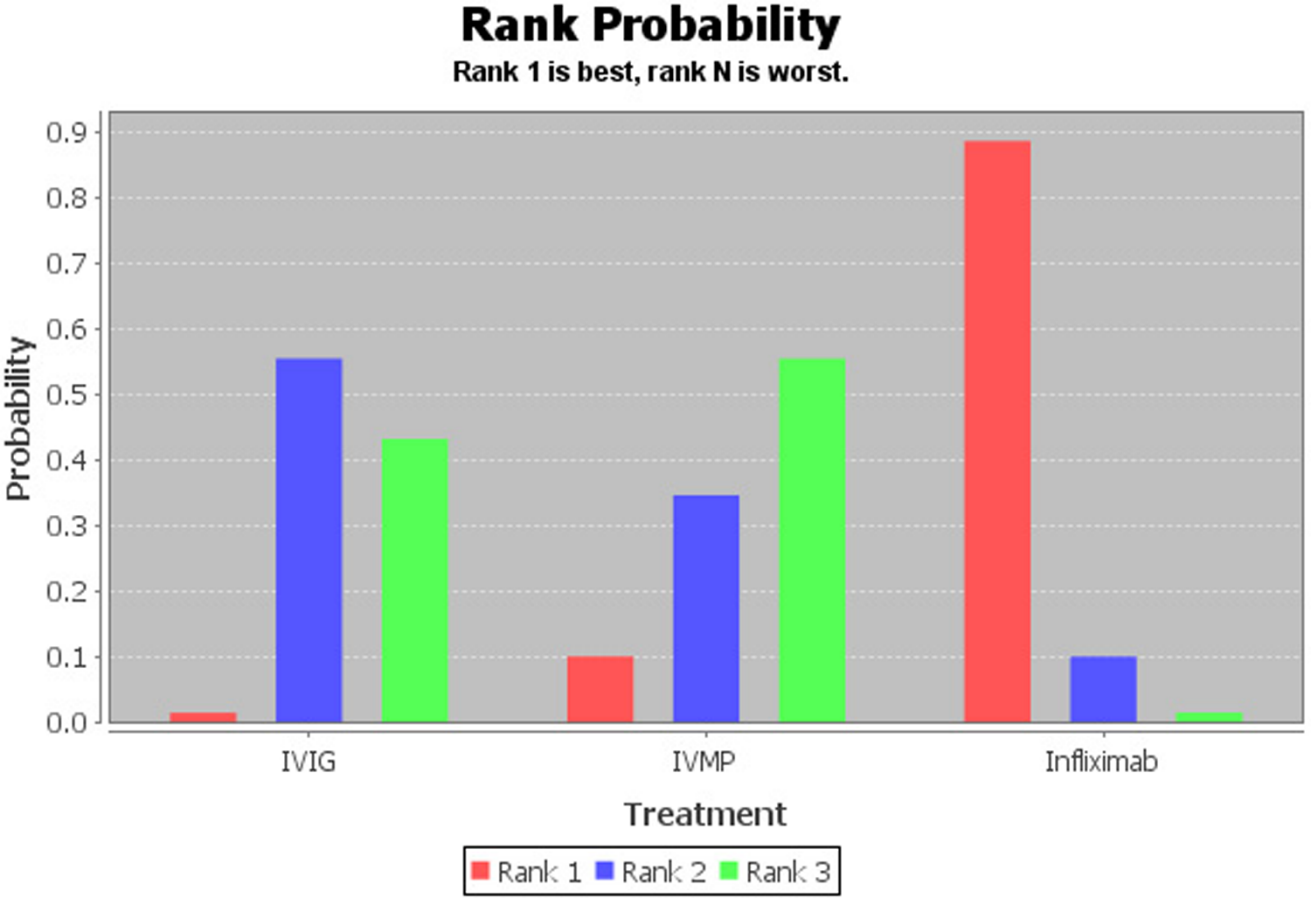
Rank probability graph of drugs. The values are given as the probability of each treatment occupying a position. Ranking 1 is the best therapy (more likely to lead to antipyretic effects) and the last one is the worst treatment for this outcome.

Infliximab was associated with significant antipyretic effects compared with the second IVIG infusion (2.46, 1.00–6.94, Figure 2). No significant differences were recorded between the IVMP and IVIG retreatment groups (0.92, 0.25–3.51). Furthermore, no significant differences were recorded between infliximab and IVMP (2.70, 0.53–14.42). According to the drug rankings (see Figure 3), infliximab had better antipyretic effects than the other drugs. Based on the current research results, infliximab is the best option against IVIG-resistant KD.

### AEs

All included studies reported AEs during the disease course, except for the study by Teraguchi et al. (Tables 2, 3). In summary, hepatomegaly was more likely to occur in the infliximab group. Patients undergoing a second IVIG treatment were more likely to develop haemolytic anaemia. Compared with the second IVIG infusion, IVMP treatment was more susceptible to bradycardia, hyperglycaemia, hypertension, and hypothermia.

**Table 2:** The incidence of AEs in the included studies (infliximab VS. IVIG).

AE description	Son		Tremoulet		Youn		Masaaki		Burns	
	Infliximab	IVIG	Infliximab	IVIG	Infliximab	IVIG	Infliximab	IVIG	Infliximab	IVIG
Hemolytic anemia	/	/	2	1	/	/	/	/	19	19
GI symptoms	/	/	/	/	/	/	2	3	3	4
Rash	/	/	/	/	/	/	3	0	2	5
Epistaxis	/	/	/	/	/	/	4	7	2	3
Infusion Reaction	/	/	/	/	0	5	/	/	2	1
Arthritis	/	/	/	/	/	/	/	/	2	4
Headache	/	/	2	1	/	/	/	/	1	2
URI	/	/	/	/	/	/	3	2	1	2
Hepatomegaly	6	1	/	/	/	/	/	/	/	/

URI, upper respiratory tract infammation; GI, gastro-intestinal; AEs, adverse effects; IVIG: intravenous immunoglobulin.

**Table 3:** The incidence of AEs in the included studies (IVMP VS. IVIG).

AE description	Miura		Furukawa		Ogata		Teraguchi		Wang	
** **	IVMP	IVIG	IVMP	IVIG	IVMP	IVIG	IVMP	IVIG	IVMP	IVIG
Bradycardia	6	2	3	0	2	0	/	/	5	0
Hyperglycemia	5	0	/	/	/	/	/	/	/	/
Hypertension	6	5	5	0	/	/	/	/	/	/
Hypothermia	/	/	3	0	/	/	/	/	/	/

AEs, adverse effects; IVIG: intravenous immunoglobulin; IVMP, intravenous pulse methylprednisolone.

### CAA

All included studies reported CAA, except for the study by Son et al. There were no significant differences in the risk of CAA between infliximab and the second IVIG infusion (1.34, 0.45–4.08). No significant differences were recorded between the IVMP and IVIG-retreatment groups (1.00, 0.25–2.91). Furthermore, no significant differences were observed between infliximab and IVMP (1.37, 0.31–8.29).

## Discussion

As reported in a multicentre study, IVIG-resistant cases may have CAA (18.6%), although the first adequate IVIG treatment can reduce IVIG nonresponse (23). Some optimal clinical treatments have been proposed to manage IVIG-resistant cases, among which a second IVIG infusion alone or in combination with corticosteroids, long-course corticosteroids alone, or infliximab plus pulsed therapy has been frequently selected. Continuous or relapsed fever following the initial IVIG dose, but not laboratory measurements of inflammation, has been recognised as an indicator of continuous inflammation. A consensus has been reached that additional treatments should be administered to patients with such symptoms. According to the KD guidelines of the American Heart Association (AHA), a second IVIG dose or steroid therapy is assigned a B evidence level (non-RCTs), whereas infliximab is assigned a C evidence level (expert consensus) (24). The International Society for Pharmacoeconomics and Outcome Research recommends using NMA to compare outcomes among diverse treatment modalities. Thus, a robust NMA is required to guide treatment.

Previous meta-analyses have shown that IVMP and infliximab treatments are more effective than the second IVIG dose in terms of fever resistance (8, 9). Our NMA comparing the three drugs, showed that infliximab was the best option for treating IVIG-resistant KD. The expression of tumour necrosis factor (TNF)-*α* increases among patients with acute KD, with the greatest expression being observed in patients developing CAA (24). TNF inhibitors can mitigate endarteritis and inflammation by inhibiting the adhesion of neutrophils onto endothelial cells (ECs). Infliximab, the anti-TNF-α chimeric monoclonal antibody, has been used to treat IVIG-resistant KD over the last decade. In retrospective studies from two institutions, IVIG-resistant KD cases receiving infliximab as initial retreatment showed markedly rapid fever resolution and shortened length of stay (LOS) relative to those receiving IVIG (17). Another study in 2021 compared the cost-effectiveness between infliximab and a second IVIG infusion in IVIG-resistant cases; according to the results, for 100 IVIG-resistant cases receiving 10 mg/kg infliximab treatment, US$ 824,759 was saved (25). Such decreased costs were related to a reduction in cost/dose and infusion duration, and 24-h monitoring prior to discharge, which shortened the LOS (14). Therefore, our study further confirmed the potential value of infliximab treatment in patients with IVIG-resistant KD. These results could be conducive to recommending an objective order of these treatment options in future studies and guidelines.

In severe KD cases, cardiovascular complications or manifestations have been strongly associated with the incidence and mortality in the acute phase or during long-term follow-up. As revealed by Millar et al., corticosteroid application in acute KD patients who developed CAA possibly induced aggravation of aneurysms, as well as impairment of vascular remodelling (15). As reported by the AHA, steroids only apply to paediatric patients who do not respond to ≥two IVIG infusions for treating continuous fever (26). However, according to previous meta-analyses, infliximab, second IVIG, and IVMP were not significantly different in CAA prevention (8, 9). Similar results were obtained in this study. These drugs may suppress cytokine generation, which is important for reconstructing the affected coronary artery wall (17). It is necessary to further investigate the long-term coronary artery outcomes among treated KD cases and to estimate coronary artery endothelium function in KD cases.

Certain limitations should be noted in this work. First, many articles included in this study were observational RCTs, which may have led to an increased risk of heterogeneity. Second, only infliximab (a TNF inhibitor) was used in every enrolled study, making it impossible to assess the efficacy of additional TNF inhibitors in IVIG-resistant KD. Third, our enrolled articles were collected from published literature, and some unpublished articles might have been missed. Finally, although no significant statistical or clinical heterogeneity was observed across the included studies, potential bias existed because the literature is limited. Most included studies did not completely evaluate the postretreatment incidence of CAAs in patients with IVIG-resistant KD after short-term follow-up. Therefore, large, homogeneous, randomised clinical trials with long follow-up periods are required.

Infliximab was the best options against IVIG-resistant KD, respectively. In addition, IVMP, infliximab and second IVIG infusion have not significant differences of prevent CAA in IVIG-resistant KD patients. More studies will need to be conducted to evaluate the different drug regimens of IVIG-resistant KD.

## Data Availability

The original contributions presented in the study are included in the article, further inquiries can be directed to the corresponding author.
